# WOX11-mediated plant resilience: nematodes cut and adventitious lateral roots surge

**DOI:** 10.1093/plphys/kiae065

**Published:** 2024-02-06

**Authors:** Alaeddine Safi

**Affiliations:** Assistant Features Editor, Plant Physiology, American Society of Plant Biologists; Department of Plant Biotechnology and Bioinformatics, Ghent University, B-9052 Ghent, Belgium; VIB Center for Plant Systems Biology, B-9052 Ghent, Belgium

Plant survival and performance heavily rely on the postembryonic development of the root system. Roots secure anchorage to the soil, facilitate water and nutrient absorption, and engage in all kinds of interactions with the rhizosphere. Seed plants have developed the ability to dynamically adjust their root architecture to cope with fluctuating soil conditions (Morales-Herrera et al. 2023). This plasticity is achieved by remodeling the growth of the primary root and the development of secondary roots.

Based on their location and the mechanism behind their formation, secondary roots are categorized into either lateral roots or adventitious lateral roots. On the one hand, genuine lateral roots emerge acropetally along the primary root in a regular spacing pattern, following periodic oscillations of auxin signaling (Chen et al. 2021). Auxin, a central player among other endogenous rooting signals, activates AUXIN RESPONSE FACTOR (ARF)7 and ARF19, which directly induce the expression of LATERAL ORGAN BOUNDARIES DOMAIN *(LBD)16* and other related *LBD* transcription factors (Okushima et al. 2007).

On the other hand, adventitious lateral roots, born in response to external cues such as tissue damage, do not follow the aforementioned acropetal pattern nor the regular intervals and can appear in between already existing lateral roots (Sheng et al. 2017). Similar to the lateral root formation process, adventitious lateral root development requires auxin-dependent *LBD16* expression. However, this pathway is not mediated by ARF7/19 but by WUSCHEL-RELATED HOMEOBOX (WOX)11 and WOX12 (Liu et al. 2014). These transcription factors (WOX11/12) are induced by wounding-triggered jasmonate-dependent auxin accumulation. Once activated, WOX11/12 promote the expression of *LBD16* and other *WOX* genes (*WOX5/7*) to mediate tissue repair and regeneration mechanisms leading to the de novo formation of secondary roots close to the cutting area (Sheng et al. 2017).

One common factor leading to root damage is the presence of plant parasitic nematodes (e.g. cyst nematodes) in the soil that impose severe biotic stress on plants, leading to substantial crop yield losses and posing a threat to food security (Ali et al. 2017).

In this issue of *Plant Physiology*, Willig et al. (2024) shed more light on various aspects of plant adaptation to cyst nematode infection via WOX11 transcription factor.

Endoparasitism by cyst nematodes causes root architectural changes in infested plants such as primary root inhibition and de novo formation of secondary roots (Guarneri et al. 2023). To investigate the identity of these nematode-induced secondary roots, the authors inoculated the lateral root-deficient *arf7/19* double mutant with the beet cyst nematode *Heterodera schachtii* (Willig et al. 2024). Although *arf7/19* plants are typically unable to form genuine lateral roots, *H. schachtii* succeeded in inducing secondary roots within the penetration site. Remarkably, overexpressing a dominant-negative version of the *WOX11* gene in the *arf7/19* mutant background (*WOX11-SRDX/arf7/19*) abolished any secondary root formation, meaning that these de novo formed roots qualify as damage-induced WOX11-dependent adventitious lateral roots (Fig.). For further confirmation, the expression of *LBD16*, the key player in root branching, was monitored. *LBD16* expression was induced in both lateral root primordia and nematode infection sites in wild-type plants. However, *WOX11-SRDX* dominant-negative lines failed to express *LBD16* at the infection zones but did express it at lateral root primordia. Together, these observations suggest that *H. schachtii* invasion activates the WOX11_LBD16 pathway to stimulate adventitious lateral root formation (Fig.) (Willig et al. 2024).

**Figure.**
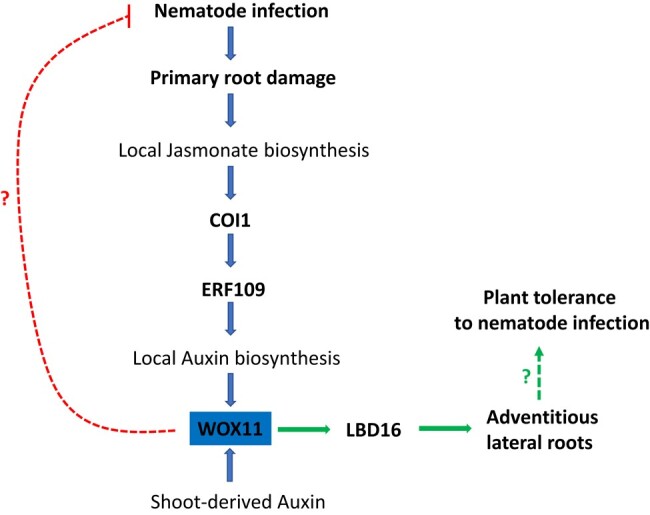
Proposed model showing the central role of WOX11 in mediating plant tolerance to cyst nematodes. Once the primary root is damaged by nematodes, jasmonate accumulates within the infection zone, leading to local auxin biosynthesis through the COI1-ERF109 pathway. Local auxin accumulation activates WOX11, which in turn induces *LBD16* expression to trigger adventitious lateral root formation near the cut zone. The de novo emerged secondary roots help the plant to overcome the damage and to compensate for the nematode-induced inhibition of primary root growth. WOX11 was also shown to decrease plant susceptibility to nematode infection. However, the mechanism behind this reduced susceptibility is still to be revealed. Green and red arrows highlight the main focus of this study. Text formatted in bold are the results of this study; the rest are from previous works. Figure adapted from Willig et al. (2024).

Using genetic analyses, Willig et al. (2024) claimed that the nematode-induced WOX11_LBD16 pathway relies on wounding-triggered jasmonate production, which activates ETHYLENE RESPONSIVE FACTOR (ERF)109 transcription factor via the CORONATINE INSENSITIVE (COI)1 receptor. Subsequently, ERF109 can trigger local auxin accumulation by directly activating auxin biosynthesis genes, leading to the upregulation of *WOX11* expression at nematode invasion sites (Fig.).

Because nematodes impede the primary root growth during plant endoparasitism, the authors proposed that adventitious lateral root formation is a way to compensate for this primary root inhibition. Indeed, the total length of the secondary roots of nematode-infested plants decreased significantly in *WOX11-SRDX* compared to wild-type, while both lines exhibited no difference in the primary root length. Moreover, the number of secondary roots emerging from infested plants positively correlates with the success rate of plant infection. However, in the plants expressing the dominant negative *WOX11-SRDX*, increased levels of plant infection did not cause increased levels of secondary roots. These findings suggest that WOX11 is triggered after *H. schachtii* infection, yielding more secondary roots as a compensation mechanism for the inhibited growth of the primary root, and that this compensation is correlated to the damage levels (Fig.) (Willig et al. 2024).

*WOX11-SRDX* lines are affected not only in adventitious lateral root formation but also in other developmental aspects as they exhibited significantly smaller rosettes compared to wild-type plants when infected with nematodes. Therefore, WOX11 plays an important role in plant tolerance to nematode infections. Interestingly, WOX11 is also crucial in restricting plant susceptibility to *H. schachtii* invasion, as *WOX11-SRDX* roots had more endoparasites than wild-type when incubated with the same number of nematodes (Fig.) (Willig et al. 2024).

In summary, Willig et al. (2024) propose a module in which nematode-triggered secondary roots have an adventitious lateral root identity driven by the WOX11 but not the ARF7/19 pathway. Furthermore, they positioned WOX11 as a central regulator in response to nematode peril as it not only reduces seedling vulnerability to infection but also increases plant tolerance after being infested (Fig.).

Further mysteries remain to be investigated such as the role of reactive oxygen species (ROS) in this pathway, especially since ROS are involved in root system architecture plasticity after cyst nematode infection (Willig et al. 2022). Additionally, WOX11/12 orthologs were found to modulate ROS levels and mediate abiotic stress tolerance in several plant species (Wang et al. 2021). One may inquire whether WOX11-dependent biotic and abiotic stress tolerance mechanisms follow the same pathway or diverge at some point. It would be of interest also to explore whether the WOX11-centered pathway is involved in the response to other plant parasitic nematodes, particularly root-knot nematodes that can target almost all vascular plant species. Because several major crops worldwide are affected by nematode invasions, investigating the cross-species conservation of the WOX11-mediated tolerance pathway is a first step toward crop protection and food production security.

## Dive Curated Terms

The following phenotypic, genotypic, and functional terms are of significance to the work described in this paper:

ARF19 Gramene: AT1G19220
ARF19 Araport: AT1G19220

WOX12 Gramene: AT5G17810
WOX12 Araport: AT5G17810

CORONATINE CHEBI: CHEBI:80730

seed AmiGo: PO:0009010

COI1 Gramene: AT2G39940
COI1 Araport: AT2G39940

ARF7 Gramene: AT5G20730
ARF7 Araport: AT5G20730
